# The Role of Extracellular Vesicles in Aging and Disease

**DOI:** 10.3390/ijms241813739

**Published:** 2023-09-06

**Authors:** Consuelo Borrás, Cristina Mas-Bargues

**Affiliations:** Freshage Research Group, Department of Physiology, Faculty of Medicine, University of Valencia, Centro de Investigación Biomédica en Red Fragilidad y Envejecimiento Saludable-Instituto de Salud Carlos III (CIBERFES-ISCIII), INCLIVA, 46010 Valencia, Spain; cristina.mas@uv.es

Cells are exposed to various internal and external factors that can cause damage over time. This damage can result from exposure to harmful chemicals, radiation, inflammation, genetic mutations, and even normal cellular processes like metabolism [1,2]. As cells accumulate this damage, their ability to function optimally and maintain their physiological state becomes compromised.

These cells communicate and cooperate to maintain the overall health and functionality of the tissue and, by extension, the entire organ. Cellular communication plays a crucial role in the aging process, as it influences how cells interact, adapt to changing conditions, and contribute to the overall function of tissues and organs. Aging is a complex biological phenomenon involving a multitude of interconnected processes, and cellular communication is integral to these processes [3]. Understanding these communication pathways and their role in aging could potentially lead to interventions promoting healthy aging and extending lifespan.

Extracellular vesicles (EVs) are small, membrane-bound structures released by cells into the extracellular environment. They play a significant role in cellular communication by transporting various molecules, including proteins, lipids, and nucleic acids, between cells [4]. EVs serve as a means for cells to exchange information and influence the behavior of neighboring or distant cells. They enable long-distance signaling and are involved in various physiological and pathological processes. Understanding the mechanisms of EV-mediated communication has implications for both our understanding of basic biology and the development of innovative diagnostic and therapeutic strategies. Extracellular vesicles (EVs) have been identified as agents capable of influencing the aging process by rejuvenating the cellular environment and fostering tissue renewal [5]. As a result, EVs hold significant promise as therapeutic tools, particularly in the realm of aging and various ailments.

Nevertheless, there is a considerable journey ahead before EVs can be effectively employed in clinical settings. It is imperative to enhance the standardization of methodologies, ensure reproducibility, and facilitate data comparison, all of which are critical aspects for advancing EV-based treatments.

To address these challenges, this Special Issue covers topics related to the role of extracellular vesicles in cellular communication, their association with various health conditions (hypertension, COPD, IPF, and acute kidney injury), their potential diagnostic and therapeutic applications, and the analysis of their profiles and cargo in different tissues and biological fluids.

Specifically, Juliana Pena Lopez et al. [6] focus in their review on spontaneous hypertension in aged male mice and how lipid profiles of urinary extracellular vesicles change during inactive and active phases. The study’s objective is to analyze the differences in lipid profiles of urinary EVs collected from aged mice during inactive and active phases. The study also explores whether these EVs influence the density of lipid rafts in specific cells (mpkCCD principal cells) and their potential impact on blood pressure regulation. The results suggest that an inhibitor of the epithelial sodium channel (ENaC) can reduce systolic blood pressure in aged male mice during both inactive and active phases. They identify distinct lipid differences between EVs from the two phases, particularly phosphatidylethanolamine plasmalogens, which are enriched in active phase EVs. These active phase EVs also appear to enhance ENaC activity in recipient cells, potentially due to increased ENaC alpha protein distribution to lipid raft fractions. This research implies that the presence of bioactive lipids in EVs released during the active phase of aged mice might influence blood pressure regulation in an age-dependent manner.

The manuscript by Gagandeep Kaur et al. [7] discusses the differences in exosomal miRNA profiles between patients with respiratory disorders such as COPD (chronic obstructive pulmonary disease) and IPF (Idiopathic Pulmonary Fibrosis). Despite extensive research on exosomal miRNAs in various diseases, there is limited knowledge about the miRNA content of bronchoalveolar lavage fluid (BALF) and lung tissue-derived exosomes in COPD and IPF. The study aimed to compare the miRNA profiles of exosomes from BALF and lung tissue in healthy non-smokers, smokers, and patients with COPD or IPF. The results showed that BALF-derived exosomes were smaller in size and had lower yield than lung tissue-derived exosomes. Notably, specific miRNAs were found to be differentially expressed in the exosomes of COPD and IPF patients. One miRNA was differentially expressed in lung-derived exosomes in COPD patients compared to healthy non-smokers. In IPF patients, a larger number of miRNAs were differentially expressed in lung-derived exosomes compared to non-smoking controls. In conclusion, the study identified lung-specific miRNAs associated with these chronic lung diseases, suggesting their potential as biomarkers or therapeutic targets for COPD and IPF.

The group of Borrás et al. [8] highlights the potential use of mitochondrial extracellular vesicles as biomarkers or therapeutic tools, suggesting their relevance to various conditions. Mitochondria play a critical role in cellular functions such as metabolism, ROS production, and apoptosis. Cellular quality control mechanisms exist to prevent the accumulation of damaged mitochondria, which can lead to the release of mitochondrial extracellular vesicles (MitoEVs). These MitoEVs carry various components, including mtDNA, rRNA, tRNA, and respiratory chain protein complexes. Notably, larger MitoEVs can transport entire mitochondria. Macrophages are involved in the engulfment of MitoEVs, leading to a process of outsourced mitophagy. Recent findings have revealed that MitoEVs can also contain healthy mitochondria, which can rescue stressed cells by restoring mitochondrial function. This transfer of functional mitochondria through MitoEVs has the potential to be used as disease biomarkers and therapeutic tools. The review discusses the emerging concept of mitochondria transfer via extracellular vesicles and highlights the current clinical applications of MitoEVs.

The text by Tomaso Neri et al. [9] emphasizes the emerging role of extracellular vesicles in COPD and their detection in various biological fluids. The most important message of this text is that chronic obstructive pulmonary disease (COPD) involves complex cellular and molecular mechanisms that are not yet fully understood. Inflammatory cells, cytokines, chemokines, and other unidentified factors play crucial roles. The disease is characterized by chronic local and systemic inflammation, lung aging, and cellular senescence, which contribute to its development and progression. Extracellular vesicles (EVs), released by various cells as microvesicles and exosomes in biological fluids, are significant in intercellular communication and are emerging as important players in the pathobiological processes of aging and chronic diseases, including COPD. The review discusses the increasing recognition of EVs as biomarkers in COPD and their potential involvement in disease mechanisms. Additionally, the text briefly covers the potential therapeutic applications of EVs as targets and agents in COPD treatment.

Finally, the manuscript by Maja Kosanović et al. [10] explores the potential of extracellular vesicles as a therapeutic avenue for renal repair and regeneration in cases of acute kidney injury (AKI). It is a significant clinical issue characterized by a sudden decline in renal function and high morbidity and mortality rates. Despite current efforts, no established treatments effectively address this condition. Extracellular vesicles have emerged as important mediators of intercellular communication within the kidney. EVs hold potential not only as diagnostic tools but also as powerful agents in regenerative medicine and therapeutic intervention in experimental models of AKI. The review provides an overview of EVs, highlighting their biological significance and functional roles in kidney cell communication. The versatile roles of EVs in crucial pathophysiological mechanisms contributing to AKI are discussed, along with a detailed exploration of the renoprotective effects of EVs from various sources in the context of AKI. The review also delves into the mechanisms by which EVs operate in AKI and offers insights into their potential clinical translation for AKI treatment.

In conclusion, this Special Issue focuses on the role of extracellular vesicles in cellular communication, their relevance to pathophysiological conditions (including hypertension, COPD, IPF, and acute kidney injury), their potential utility as biomarkers and tools for therapy, and their investigation within diverse biological fluids and tissues.
